# Quality improvement and workplace wellbeing capacity and capability in Aotearoa New Zealand emergency departments. A nationwide mixed methods survey

**DOI:** 10.1093/intqhc/mzaf073

**Published:** 2025-08-06

**Authors:** Mike Nicholls, Natalie Anderson, Rebecca Jarden, Vanessa Selak, Chris Frampton, Stuart R Dalziel

**Affiliations:** Department of Paediatrics, Faculty of Medicine and Health Sciences, The University of Auckland, Private Bag 92019, Auckland Mail Centre, Auckland, 1142, New Zealand; Adult Emergency Department, Te Toka Tumai Auckland City Hospital, Private Bag 92 024, Auckland Mail Centre, Auckland, 1142, New Zealand; Adult Emergency Department, Te Toka Tumai Auckland City Hospital, Private Bag 92 024, Auckland Mail Centre, Auckland, 1142, New Zealand; School of Nursing, Faculty of Medical and Health Sciences, The University of Auckland, Private Bag 92019, Auckland Mail Centre, Auckland, 1142, New Zealand; Department of Nursing, The University of Melbourne, School of Health Sciences Melbourne, Victoria, 3010, Australia; Epidemiology and Biostatistics Department Faculty of Medical and Health Sciences , The University of Auckland, Private Bag 92019, Auckland, 1142, New Zealand; Department of Medicine, Christchurch School of Medicine and Health Sciences, University of Otago, PO Box 4345, Christchurch, 8140, New Zealand; Department of Paediatrics: Child and Youth Health, The University of Auckland, Private Bag 92019, Auckland, 1142, New Zealand; Children’s Emergency Department, Starship Children’s Health, Private Bag 92024, Auckland, 1142, New Zealand

**Keywords:** Burnout, Psychological; quality improvement; Emergency Service, Hospital; Surveys and Questionnaires

## Abstract

**Background:**

Healthcare workers in emergency departments (EDs) need to function optimally to deliver high-quality healthcare. High levels of burnout negatively impact these workers’ delivery of quality care to their patients. Quality improvement and workplace wellbeing programmes may simultaneously improve quality of care and burnout. Global recommendations for strengthening quality improvement and workplace wellbeing are increasingly available for healthcare services. Understanding organizational capacity and capability for improvement and wellbeing is an important first step in co-designing change. This study sought to assess the capacity and capability for quality improvement and workplace wellbeing initiatives in New Zealand EDs.

**Methods:**

In a convergent mixed methods study, a cross-sectional online survey was conducted in August–October 2022. Quality and/or workplace wellbeing leaders were invited to participate from each of the 35 EDs across New Zealand. Questions were based on a range of published tools described below. Participants were invited to contribute further details in free-response items. Quantitative data were analysed using descriptive statistics, and qualitative data were analysed thematically, then data were integrated.

**Results:**

Sixty leaders (30 medical, 29 nursing, 1 other) representing 29/35 (83%) of EDs responded. Overall quantitative results demonstrated considerable gaps between reported and best-possible capacity and capability for quality improvement and workplace wellbeing. Institute for Healthcare Improvement Self-Assessment Tool mean score was 2.4 (maximum 5; SD 0.8); Improvement Readiness scale mean score was 3.1 (maximum 5; SD 0.9); a positive improvement climate was identified in 13/60 (22%) EDs; Australasian College for Emergency Medicine Quality Standards Implementation Toolkit measures, means were 51 (maximum 100; SD 21) for quality improvement and 46 (SD 20) for wellbeing; Shanafelt’s Era of workplace wellbeing mean score was 3.1 (maximum 5; SD 0.6); for 11/59 (19%) respondents, none of the measures of the Joy in Medicine Health System Recognition Program applied to their ED. Three qualitative themes were identified: ‘work-related frustration and distress’; ‘desire, motives, actions, effort to make things better’; and the ‘interdependence of high-quality care and workplace wellbeing’. Data integration strengthens understanding of quantitative and qualitative results and analyses, which were congruent throughout the study.

**Conclusion:**

Capacity and capability for quality improvement and workplace wellbeing across all survey items were generally perceived to be low. Participants expressed perceptions of healthcare worker distress and wasted efforts. Addressing and building capacity and capability in both quality improvement and workplace wellbeing initiatives is a potential first step in improving quality of patient care and healthcare worker wellbeing.

## Introduction

The interdependence of the ability to deliver high-quality healthcare and the wellbeing of healthcare workers (HCWs) has been acknowledged in quality frameworks such as the ‘quadruple aim’ [1]. HCWs need to function optimally in order to deliver high-quality healthcare for patients and healthcare systems worldwide [1–3]. This is particularly true in emergency departments (EDs), which operate as semiautonomous units within a complex healthcare system. While EDs are connected to and share similarities with other healthcare units, each ED is unique, and HCWs may reasonably believe that they may have some influence over departmental priorities, activities, and culture. However, 55% of New Zealand (NZ) ED HCWs surveyed in 2020 reported work-related burnout [4]. Numerous factors impact workplace wellbeing and burnout [1, 5], and there may be many potential solutions. In addition to individual-focussed approaches [6, 7], other approaches use system-level scientific improvement methods such as quality improvement (QI) [8].

In addition to the content of any improvement initiative, how, and within what *context* QI is implemented is vital to success [9, 10]. Organizational *capacity* for improvement refers to having the right number and level of staff members who are actively engaged to take action, while *capability* is staff members having the confidence, knowledge and skills to lead the change [10].

Although tools to assess capacity and capability to undertake healthcare QI are widespread, there is no well-validated, consistent approach to assessment at the unit (department) level [10, 11]. One approach employs ‘maturity models’, ‘assessment approaches that examine the degree of formality and optimization of processes, from ad-hoc practices, to pro-active optimization (of) processes over time’ [11]. The Institute of Healthcare Improvement (IHI) Improvement Capability Self-Assessment Tool [12] is an example. Similarly, a ‘maturity’ lens can also be used for HCW wellbeing, for example, Shanafelt’s ‘eras’ of wellbeing [13].

As a necessary precursor to optimizing HCW wellbeing and the quality of care they deliver, this study sought to answer the question what are the capacity and capability for QI and workplace wellbeing in NZ EDs. To the authors’ knowledge, this had not previously been answered.

## Methods

This nationwide online cross-sectional survey was conducted in NZ EDs with an annual census of >5000 patients (*n* = 35). Participant inclusion criteria were: ED Clinical Director or Nurse Unit Manager; or medical, nursing or other lead of an ED quality group and/or wellbeing group (or similar); or senior ED doctor or nurse with involvement in QI or workplace wellbeing. Participants were excluded if they were not a permanent ED employee.

Given the study was an assessment of all NZ EDs, no formal power calculation for sample size was required. Two participants were sought from each eligible ED, so a maximum of 70 survey participants were expected. Potential participants were identified for recruitment using authors’ contact lists and those contacts were encouraged to forward the invitation email to their own contacts. An invitation with an electronic survey link was emailed on 29 August 2022. Weekly reminders were sent for 4 weeks. Coffee beans were provided as incentive and gratitude for participation. The last response was received on 1 October 2022.

The survey included questions about participant age, ethnicity, professional group, leadership and years of ED experience, attendance at formal QI courses, involvement in workplace wellbeing and QI projects, and for which ED they were responding. As no single valid, reliable tool to assess QI and workplace wellbeing capacity and capability in NZ EDs was identified, a bespoke questionnaire was developed, including QI questions from four sources:

- IHI Self-Assessment Tool [12]; includes 13 statements related to six domains. Participants rated their ED on a five-point Likert Scale from 1 (just developing) to 5 (exemplary).
- Improvement Readiness (IR) scale [14], one component of the SCORE (Safety, Communication, Operational Reliability, and Engagement) survey [15]. Participants rated their ED on five statements on a five-point Likert Scale from 1 (disagree strongly) to 5 (agree strongly). EDs rated as having a mean of ≥4 were considered to have a positive IR climate.
- Australasian College for Emergency Medicine (ACEM) Quality Standards Implementation Toolkit [16]. Participants rated their ED on a 0–100 sliding scale from 0 (ED does not fulfil standard at all) to 100 (ED fulfils standard) on 15 standards related to QI.
- A single supplementary question ‘How well integrated with the hospital Performance or Quality Improvement team is your ED team?’ composed by the lead investigator, rated as per the ACEM standards.

Wellbeing questions were from three sources:

- Shanafelt’s ‘Era’ of workplace wellbeing [13]. Comprises 17 statements. For 14 of these participants rated their ED on a five-point Likert scale from 1 (era of distress) to 5 (wellbeing 2.0). Three of the 17 statements referred to organizational mindset and participants were asked if these applied to their ED (yes/no).
- Joy in Medicine Health System Recognition Program [17]. Participants were asked to rate their ED ‘bronze’, ‘silver’, or ‘gold’, or ‘not applicable/other’ against statements covering five competencies.
- ACEM Quality Standards Implementation Toolkit [16]. Twenty standards related to wellbeing were rated, as described above.

Opportunities were provided throughout the survey for participants to voice more detail using open-ended statements. Quantitative assessment was prioritized, but because more insight could be gained from analysing both quantitative and qualitative data than either alone, a convergent mixed methods approach was used to analyse data and integrate results [18].

M.N. made minor adjustments to the original tools, to more appropriately suit the context, and enable quantitative assessment. The survey was previewed by co-authors (except C.F.) and piloted by a small group of NZ ED wellbeing investigators to check usability, relevance, and understanding, resulting in minor wording changes. Supplementary Files S1 and S2 provide further details about the process and rationale for question selection and modifications, and all survey questions.

Data were collected anonymously using REDCap (REDCap 9.4.1, Vanderbilt University, Nashville, TN) software, and managed in compliance with University of Auckland standards. Quantitative data were analysed and summarized by a senior statistician using SPSS Statistics for Windows (V28.0., IBM Corp, Armonk, NY). Missing data were managed as detailed in the results section. Qualitative data were analysed inductively, guided by six-phase reflexive thematic analysis (TA) [19] using NVivo software (V14, Lumivero, Denver, CO) to organize data. Qualitative data analysis was led by M.N., an emergency physician doing his first TA research project, facilitated by discussions with and oversight by N.A., an emergency nurse and experienced postdoctoral qualitative researcher. Integration of quantitative and qualitative results [18] was led by M.N. facilitated by discussion with N.A. and other co-authors.

Data collection followed Auckland Health Research Ethics Committee approval. All participants provided informed consent prior to commencing the survey. Research is reported using appropriate reporting guidelines [20, 21].

## Results

Sixty staff representing 29/35 (83%) of NZ EDs responded. Compared to those participating, most EDs without participants (*n* = 6) were in the South Island (*n* = 5) and had a smaller median annual patient census (10 081 vs. 32 962; 2018 data). While most EDs were represented by two participants as intended (24/29; 83%), two EDs had a single participant, two EDs had three participants, and one had four participants.

Participant characteristics are outlined in Table 1. Half were doctors, most were female and NZ European. Three-quarters had a minimum of 11-year ED experience, reflecting the seniority of the cohort. Almost two-thirds (37/60; 62%) had attended one or more formal QI training courses, while 15% (9/60) had been involved with six or more wellbeing projects.

**Table 1.** Participant characteristics

		*n* (%)
Gender	Female	47 (78)
Age (years)	25–34	5 (8)
	35–44	18 (30)
	45–54	22 (37)
	55–64	15 (25)
Ethnicity (prioritized)	Māori	4 (7)
	Pacific Peoples	3 (5)
	Asian	3 (5)
	NZ European	45 (75)
	Other	5 (8)
Years of ED experience	1–5	6 (10)
	6–10	9 (15)
	11–15	14 (23)
	16–20	16 (27)
	>20	15 (25)
Profession	Nurse	29 (48)
	Doctor	30 (50)
	Other	1 (2)
Number of QI projects^a^ involved with		
	0	4 (7)
	1–5	22 (37)
	6–10	14 (24)
	11–15	8 (14)
	16–20	5 (8)
	>20	6 (10)
Number of wellbeing projects involved with^b^^,^ ^d^		
	0	14 (24)
	1–5	35 (59)
	6–10	7 (12)
	11–15	2 (3)
	16–20	0
	>20	1 (2)
Number who had done at least one of the providers’ courses^b^:		
	HQSC^c^	13 (22)
	IHI	10 (17)
	University	21 (35)
	Other	24 (40)

a Question answered by 59/60 respondents.

b Some participants attended courses by more than one provider, so total >100%.

c HQSC, Health Quality and Safety Commission|Te Tāhū Hauora.

d Question answered by 59/60 respondents

Quantitative results related to QI are displayed in Tables 2 and 3 (Statements 1–16). Overall means of the IHI and the IR scales were 2.4 (SD 0.8) and 3.1 (SD 0.9), respectively. Just 13 of 60 participants (22% 95% CI 13%–34%) rated their ED as having a positive IR climate (overall IR scale mean of 4 or more). Very few domains were rated 5 (exemplary, or agree strongly) in either scale. In the IHI scale the ‘leadership’ domain was the most highly rated, and ‘workforce’ rated lowest. ‘Utilizes suggestions’ domain was most highly rated in the IR scale with ‘protected’ rated lowest.

**Table 2.** QI capacity and capability in NZ ED measured by (a) IHI Improvement Capability Self-Assessment Scale and (b) IR Scale

(a) IHI Improvement Capability Self-Assessment Scale, *n* (%)							
	Just beginning	Developing	Making progress	Significant impact	Exemplary		
**Domain**	1	2	3	4	5	*n* ^a^	Mean (SD)
**Leadership:** The capability of the leadership of the department to set clear improvement goals, expectations, priorities, and accountability and to integrate and support the necessary improvement activities within the ED	10 (17)	18 (30)	19 (32)	13 (22)	0 (0)	60 (100)	2.6 (1.0)
**Results:** The capability of a department to demonstrate measurable improvement across all areas	12 (20)	19 (32)	27 (45)	2 (3)	0 (0)	60 (100)	2.3 (0.8)
**Resources:** The capability of a department to provide sufficient resources to establish improvement teams within the department and to support their ongoing work and success	10 (17)	23 (38)	18 (30)	9 (15)	0 (0)	60 (100)	2.4 (0.9)
**Workforce:** The capability of a department to organize its workforce to encourage and reward active participation in improvement work, clearly define and establish improvement leadership roles, and ensure that job descriptions include a component related to improvement work	15 (25)	19 (32)	20 (33)	6 (10)	0 (0)	60 (100)	2.3 (1.0)
**Data:** The capability of a department to establish, manage, and analyse data for improvement in a timely and routine manner to meet the objectives and expected results of the department’s improvement plan	11 (18)	21 (35)	18 (30)	7 (12)	3 (5)	60 (100)	2.5 (1.1)
**Knowledge and Competence:** The capability of a department to obtain and execute on the skills and competencies required to undertake improvement throughout the department	20 (33)	13 (22)	16 (27)	10 (17)	1 (2)	60 (100)	2.3 (1.2)
						Overall mean	2.4 (0.8)

(b) IR Scale, *n* (%)							
Domain	Disagree strongly		Neutral		Agree strongly		
*The improvement environment in this work setting…*	1	2	3	4	5	Total respondents	Mean (SD)
** Utilizes suggestions: ** utilizes input/suggestions from the people who work here.	2 (3)	11 (18)	10 (17)	30 (50)	7 (12)	60 (100)	3.5 (1.0)
**Integrates lessons:** integrates lessons learned from other work settings.	2 (3)	16 (27)	15 (25)	25 (42)	2 (3)	60 (100)	3.2 (1.0)
**Fixes defects:** effectively fixes defects to improve the quality of the work we do.	5 (8)	13 (22)	24 (40)	16 (27)	2 (3)	60 (100)	3.0 (1.0)
**What we do well:** allows us to gain important insights into what we do well.	3 (5)	12 (20)	19 (32)	24 (41)	1 (2)	59 (100)	3.1 (0.9)
**Protected:** is protected by our local management.	13 (22)	11 (19)	16 (28)	17 (29)	1 (2)	58 (100)	2.7 (1.2)
						Overall mean	3.1 (0.9)

a *n*, total respondents.

**Table 3.** QI and workplace wellbeing capacity and capability in NZ EDs measured by ACEM standards^a^

Statement number and explanation		*n* ^b^	Mean (SD)
1	**Domain: Cultural** The ED team utilizes Māori health models to inform clinical practice e.g. the *Meihana* Model, based on the Māori health framework *Te Whare Tapa Wha*, to support ED staff to gain a broader understanding of Māori patients’ presentations, and guide clinical assessment and treatment/intervention with Māori clients and whānau	56	45 (31)
2	**Domain: Administration** The composition of the ED team supports the necessary clinical and clinical support functions of the ED, including quality assurance, education provision, administration, research, maintenance, security, cleaning	51	55 (26)
3	**Domain: Administration** The ED team engages with hospital administration and other hospital departments to enhance communication and accountability, and improve quality, safety, and efficiency of care	51	61 (26)
4	**Domain: Administration** The ED team has established actions to work collaboratively with hospital executive and inpatient units	52	63 (26)
5	**Domain: Administration** The ED team has access to consumer representatives to engage in quality assurance and improvement activities	51	41 (27)
6	**Domain: Administration** The ED team demonstrates a capacity to learn from everyday successes as well as from adverse events	50	64 (27)
7	**Domain: Professionalism** Staff are empowered and supported to lead	51	54 (29)
8	**Domain: Professionalism** The ED team ensures each team member has the opportunity to debrief following a complex or stressful situation	55	52 (31)
9	**Domain: Professionalism** Debriefing processes address clinical and emotional issues	50	58 (28)
10	**Domain: Education and Training** The ED team ensures that the provision of inter-professional learning opportunities results in consistent work practices and expectations amongst the ED team	51	54 (25)
11	**Domain: Education and Training** The ED team has access to relevant nontechnical skills training that enables the ED team to provide high quality care to patients including communication skills, graded assertiveness, human factors, patient safety, continuous QI, teamwork	53	49 (29)
12	**Domain: Research** The ED team has a process to systematically review and implement research and evidence-based findings relevant to the ED	50	43 (27)
13	**Domain: Research** All members of the ED team are encouraged and empowered to participate in or contribute to QI	52	56 (26)
14	**Domain: Research** Training and resources are provided to the ED team to conduct QI	49	49 (28)
15	**Domain: Research** Patient involvement in QI processes is encouraged and supported	51	40 (27)
16	How well integrated with the hospital performance or QI team is your ED team?^c^	50	48 (23)
17	**Domain: Administration** The ED is designed to promote a positive environment for the ED team, patients, and whānau/carers, embracing cultural values of local communities, including Māori	52	51 (27)
18	**Domain: Administration** The ED utilizes good work design, where hazards and risks are removed or minimized and the wellbeing of workers is prioritized. Optimally, risks to workforce health and wellbeing are ‘designed out’ of the work, where possible	50	45 (25)
19	**Domain: Administration** Adequate facilities are provided for staff including change rooms, showers and toilets, secure storage, meal or break areas, office space, areas for group education, areas for breastfeeding, rest, reflection, prayer	52	42 (31)
20	**Domain: Administration** The ED team is involved in any investigation of increased turnover, including conducting exit interviews	54	30 (27)
21	**Domain: Administration** Turnover is minimized by system level interventions aimed at improving workforce experience	54	23 (24)
22	**Domain: Administration** Occupational Health and Safety representatives are supported and easy to contact	56	45 (28)
23	**Domain: Administration** Psychological safety is supported as well as physical safety	54	43 (29)
24	**Domain: Administration** The ED team advocates for the ED to be a safe and secure environment for team members	53	63 (28)
25	**Domain: Administration** The ED team ensures that team members have access to evidence-based counselling, for work-related stresses	54	68 (29)
26	**Domain: Administration** The ED team has systems in place where staff are regularly consulted in relation to how work is conducted, and departmental health and safety issues	54	44 (28)
27	**Domain: Administration** The ED has systems in place to identify, assess, and control both physical and psychological risks of harm in the workplace	53	41 (25)
28	**Domain: Administration** There are processes in place for identifying, assessing, and controlling physical, psychological, and psychosocial hazards and risks e.g. fatigue, high workloads, emotional demands, traumatic events, occupational violence, bullying and harassment, manual tasks	52	36 (25)
29	**Domain: Administration** The ED has a wellbeing policy which reflects support for the ED team as a priority	56	32 (30)
30	**Domain: Administration** The ED team monitors absences to observe for signs of stress in the workforce	54	39 (27)
31	**Domain: Administration** Rosters comply with safe working hours recommendations	50	60 (29)
32	**Domain: Administration** Staff are able to access leave entitlements including sick leave, parental leave, annual leave, professional development, carer’s leave, leave for cultural obligations and celebrations	55	69 (27)
33	**Domain: Administration** The ED team has a healthy workplace plan which encourages the pursuit of health and wellbeing for team members. This includes providing education and training in achieving a healthy lifestyle and participating in preventative and health improvement initiatives	54	43 (26)
34	**Domain: Administration** There are processes in place to monitor staff wellbeing, provide early intervention, and referral to appropriate support	55	35 (26)
35	**Domain: Professionalism** The ED team ensures that mentoring is available to all ED team members separate from supervision or appraisal processes and is responsive to the needs of the mentee	53	46 (30)
36	**Domain: Professionalism** ED leaders advocate for the psychological and physical safety and wellbeing of all team members, and work to address system factors that cause harm to the workforce	51	50 (29)

a Statements 1–16 relate to QI, Statements 17–36 relate to workplace wellbeing.

b *n*, number of respondents.

c All statements are from ACEM standards except Statement 16 from the authors.

Statements 1–16 in Table 3 relate to QI and are from the ACEM toolkit (Statements 1–15), and the authors’ single statement (Statement 16). The overall mean of these statements, requiring at least 15/16 responses (*n* = 40) to account for missing data, was 51 (SD 21). The most favourably rated statement related to learning from success (Statement 6). Statements related to patient and consumer representative involvement in QI were rated lowest (Statements 5 and 15). The statement related to Māori models of care (Statement 1) is also lowly rated 45 (SD 31).

Quantitative results for workplace wellbeing are displayed in Table 3 (Statements 17–36 from the ACEM toolkit) and Table 4. The overall mean of ACEM statements, requiring at least 18 of 20 possible responses (*n* = 48) to account for missing data, was 46 (SD 20). Statements relating to access to leave (Statement 32) and access to counselling (Statement 25) were the most highly rated. However, of concern both were only rated in the 60s, and could be considered mandatory, especially access to counselling, which is supported by legal frameworks in NZ [3] for good workplace wellbeing. Statements relating to staff turnover had the lowest rating (Statements 20 and 21), with statements related to wellbeing policy and monitoring of HCW wellbeing also lowly rated (Statements 29 and 34).

**Table 4.** Workplace wellbeing capacity and capability in NZ EDs measured by Shanafelt’s Era of workplace wellbeing

(a) Shanafelt’s Era of workplace wellbeing								
	Era of distress		Wellbeing 1.0		Wellbeing 2.0	**N/A** ^a^	***N*** ^b^	Mean (SD)
	1	2	3	4	5	0		
**1. Awareness state**	Lack of awareness: inattention to the impact of ED staff distress		Awareness: appreciation of the implications of ED staff distress and wellbeing		Action: system interventions to prevent ED staff distress			
	2 (3)	18 (31)	16 (27)	17 (29)	6 (10)	0	59	3.1 (1.1)
**2. Professional culture**	Culture of perfection		Culture of wellness		Culture of vulnerability and self-compassion			
	0	11 (20)	20 (37)	20 (37)	3 (6)	5 (9)	54	3.3 (0.9)
**3. Professional icon**	ED staff with deity (god)-like qualities		ED staff with hero-like qualities		ED staff with human qualities			
	0	1 (2)	5 (9)	14 (25)	36 (64)	3 (5)	56	4.5 (0.7)
**4. Training mindset**	‘Rites of passage’		Competency-based framework		Organizational environment that attends to both developing competence and holistically caring for ED staff			
	3 (5)	6 (10)	29 (49)	14 (24)	7 (12)	0	59	3.3 (1.0)
**5. Organization mindset**	Blame individuals for distress (personal weakness)		Appreciation that system factors cause distress but promulgate personal solutions		Address system issues through human factors engineering; evolve characteristics of organizational culture that contribute to distress			
	2 (3)	13 (22)	18 (31)	23 (39)	3 (5)	0	59	3.2 (1.0)
**6. ED staff-administrator relationship**	Mutual neglect		Adversarial: ED staff blame administrators for the problems; administrators blame staff		ED staff-administrator partnership to create solutions			
	1 (2)	6 (10)	14 (24)	28 (48)	9 (16)	1 (2)	58	3.7 (0.9)
**7. ED staff mindset**	No limits on work		Balance personal and professional life		Integration of personal and professional life including boundaries and healthy limits			
	7 (12)	23 (39)	21 (36)	7 (12)	1 (2)	0	59	2.5 (0.9)
**8. Administrator mindset** • Organizations that view the issue as a zero-sum game problem have a mindset that the only way to relieve ED staff from excessive workload and administrative burden is to shift this work to others. This framework suggests that system approaches to improve wellbeing of some ED staff would invariably worsen the wellbeing of other members of the healthcare team.								
• In a nonzero-sum game, all parties can gain.								
	Disregard of ED staff distress		‘Zero-sum game’ problem		‘Nonzero-sum game’ problem			
	10 (19)	13 (25)	9 (17)	18 (34)	3 (6)	6 (11)	53	2.8 (1.3)
**9. Funders (payers) and regulators mindset**	No attention to impact that regulations and administrative decisions have on ED staff		Awareness of impact that regulations and administrative decisions have on ED staff		Regulations and administrative decisions influenced by needs of ED staff			
	15 (27)	17 (30)	18 (32)	6 (11)	0	3 (5)	56	2.3 (1.0)
**10. Orientation to colleagues**	Isolation		Connection		Community (shared experience; care for each other, mutual support)			
	3 (5)	13 (22)	16 (28)	20 (35)	6 (10)	1 (2)	58	3.2 (1.1)
**11. Approach to individual distress**	Neglect/ignore		Treat		Prevent distress and promote professional fulfilment			
	4 (7)	14 (24)	17 (29)	21 (36)	3 (5)	0	59	3.1 (1.0)
**12. Approach to technology contributions**	Not applicable/lower relevance		Teach ED staff tips and tricks to optimize their ability to use suboptimal IT^c^	Demand better IT products from vendors	Collaborate with IT and regulatory bodies to limit suboptimal IT			
	2 (3)	17 (29)	30 (51)	4 (7)	6 (10)	0	59	2.9 (1.0)
**13 Scholarship and research focus**	Rare descriptive studies of burnout/similar in some ED groups		Limited testing of individual interventions to mitigate distress		Rigorous testing of system-level interventions to mitigate distress and to promote staff wellbeing			
	17 (34)	12 (24)	16 (32)	4 (8)	1 (2)	8 (2)	50	2.2 (1.1)
**14. Resource allocation mindset regarding staff wellness**	Ignorance of the issue	Staff well-being is a necessary cost	Return on investment	Value on investment	Staff well-being is a foundational value and core organizational strategy			
	9 (16)	27 (47)	7 (12)	7 (12)	7 (12)	2 (4)	57	2.6 (1.3)
					**Overall mean^d^**			**3.1 (0.6)**

**(b) Organizational mindset component of Shanafelt’s Era of workplace wellbeing** ^e^					
Organizational mindset:	Era of distress	Wellbeing 1.0	Wellbeing 2.0	**N/A** ^a^ **, other**	***N*** ^b^
**To focus on needs**	Institutional needs	Patient needs	Needs of people (patients, *whānau* and staff)		
	42 (71)	29 (49)	31 (53)	2 (3)	59
**Towards workplace wellbeing**	Individuals	Teams	Systems		
	32 (54)	26 (44)	25 (42)	4 (7)	59
**To solutions**	Self-care	Personal resilience	Infrastructure and leadership to advance wellbeing		
	35 (59)	42 (71)	24 (41)	3 (5)	59

a N/A, not applicable.

b *N*, number of participants that provided a valid response.

c IT, information technology.

d To account for missing data, overall mean calculated from results of those who had answered ≥13 of 14 statements (*n* = 52).

e In Table 4b participants could check as many options as they felt appropriate, so total responses for each domain are greater than number of participants.

Results from Shanafelt’s ‘Era’ of workplace wellbeing are displayed in Table 4a and b. The overall mean in Table 4a is 3.1 (SD 0.6). This indicates that, overall, participants consider that their EDs are characterized most closely with features consistent with Shanafelt’s Era 1.0. The professional icon domain is the most highly rated, with ED HCWs overwhelmingly considered to have human, rather than hero- or deity-like qualities. The research and scholarship domain rated lowest. Encouragingly, approximately half the participants checked a response consistent with Era 2.0 for each of the three statements regarding organizational mindset (Table 4b).

For each of the five individual components measured in the Joy in Medicine Health System Recognition Program [17] (Supplementary File S3), the most frequent response was that their ED did not meet a bronze, silver, or gold criteria. Very few EDs had paid positions for wellbeing and only a third had a formalized wellbeing group. Overall 11/59 (19%) respondents considered none of these criteria applied to their ED.

Taken together, quantitative results relating to QI (Tables 2 and 3) and workplace wellbeing (Tables 3 and 4) are approximately half of what may be considered ideal, implying considerable gaps between reported and best-possible capacity and capability for QI and workplace wellbeing in NZ EDs.

### Qualitative results

Three themes (Table 5) were inductively generated from the data by these insider researchers. Theme 1 highlights frustrations and distress experienced by study participants, their colleagues, and others related to working in NZ EDs. Theme 2 relates to participants’ desires and efforts to improve, at individual, group and system levels. While some worked in departments that had seemingly mature systems for improvement and workplace wellbeing, those working in poorly developed systems report wasted efforts, compounding their frustration. The interdependence of the quality of care provided and HCW wellbeing (Theme 3), and the role QI may play in improvement, was articulated by some participants, while others articulated that these were unrelated, even mutually exclusive, concepts.

**Table 5.** Qualitative assessment of QI and workplace wellbeing in NZ EDs

Theme with summary quote	Description, discussion, and relationship to study aim	Illustrative participant responses
**Theme 1. Work-related frustration and distress** ‘*It is very hard to see your team members you care about struggle*’	Working in NZ EDs can be frustrating and distressing due to many factors that arise from many sources. The degree of distress can be substantial. Participants describe working in an overly demanding environment that left little capacity to attend to improvement of care, nor to staff wellbeing. Staffing issues were especially prominent, including lack of staffing numbers, lack of staff seniority, and pressure to work extra to cover shortages. Sources of distress from system issues were numerous. Some examples include inequity between staff groups (doctors vs nurses) and rural EDs, feeling unheard by and disconnected from leaders and managers, and the lack of robust efficient process for improvement.	‘*It is very hard to see your team members you care about struggle with a large workload, short-staffing, undeliverable, unrealistic expectations for care within these constraints*’. p38 Nurse ‘*This department has some very frustrating issues with regards to process that are directly affecting patient outcomes and more importantly staff wellbeing due to the stressful workload this has caused*’. p56 Nurse ‘*until we have enough staff to be able to take annual leave, enough nurses to fill the shifts and equitable pay with other EDs across the country, adequately paid nurses, an adequate sized ED for the volume of staff and I get an office that is not also the department meeting room has 2 computers shared with 12 consultants… then the rest is tits on a bull*’. p23 Doctor ‘*there is a QIP meeting but no actual quality improvement methodology used (PDSA cycles, fishbones diagrams, goal or strategy setting). Folk in my department have no idea what those things are- look at me like I*’*m an alien and now I*’*ve fortgotten how—despite having been very well taught during my* (country) *training*’. p24 Doctor ‘*Our I.T. sucks*’. p9 Doctor ‘*There is a massive disconnect within our department in terms with regards to staff wellbeing/safety. Concerns are raised repeatedly at junior and senior level and are deflected by our management structure. This has resulted in high levels of stress, resignations and ongoing unsafe working conditions*’. p52 Nurse
**Theme 2. Desire, motives, actions, effort to make things better.** ‘*If staff choose to join in a project, they do so without any training and just make stuff up—this results in ineffective/inefficient outcomes*’	Using a range of approaches, participants want to act, and do take actions, to improve. The focus of improvements can be person-, group-, or system centred. **Subthemes: Person- and group-focused** Compassionate HCWs do their best to take action to improve things, including taking action to relieve the distress in self, colleagues, and health service users. Participants highlighted efforts targeting support at individual and group levels. **Subtheme: System-focused** While there were examples of lack of system capacity and capability to make improvement, there were examples of success with structured QI efforts. A range of effort may be required, from seemingly little to apparently massive effort. Efforts can be ad-hoc or take a more systematic approach. The lack of capacity and capability within a workplace can result in ‘effort-reward imbalance’, with substantial efforts by HCWs without the intended outcomes.	‘*Look after each other*’. p57 Nurse ‘*We are a small team that help each other out*’. p20 Doctor ‘*Senior nursing observes and intervenes quickly and supports when a staff member in distress or not coping*’. p38 Nurse ‘*social gatherings outside of work, weekly waiata* (singing) *with staff across the hospital…*’. p16 Doctor ‘*There appears to be a lack of any co-ordinated efforts to improve workplace wellbeing within our department from within our management structure. Any efforts to introduce change are driven by individuals/small groups and therefore suffer from lack of resource/support. The people attempting to drive the change often get fatigued or emotionally injured as a result*’. p52 Nurse. ‘(These EDs) *have always had a strong data-driven support for change, improvements, comms to teams*’. p38 Nurse ‘*we have zero true quality improvement processes (though I think colleagues think we do) we do have audit but no methodology for improvement after that*’. Doctor p24 ‘*Our biggest gap is we have no nurses with paid time to be involved in projects and you cannot do them without nursing input—it has to be multidisciplinary*’. p24 Doctor ‘*No quality improvement can move forward if no one is available to implement proposed changes*’. p57 Nurse ‘*If staff choose to join in a project, they do so without any training and just make stuff up—this results in in effective/inefficient outcomes as there is a lack of direction*’. p56 Nurse ‘*Staff are encouraged to undertake QI, doctors get some time to do it, nurses none, and admin none*’. p41 Nurse. *Still working on a culture change where QI is seen as everyone*’*s responsibility and they have time to engage in it*’. p12 Doctor ‘*Our QI program has been ad hoc until recently but over the past 6 months is finally being formalized and is moving in a steadily improving trajectory*’. p3 Doctor
**Theme 3. Interdependence of delivering high-quality healthcare and workplace wellbeing** ‘*QI and wellbeing support for staff are essential to providing excellent patient care and improving staff wellbeing*’	An underlying assumption for the survey was that NZ ED HCWs cannot sustain the provision of high-quality healthcare without the wellbeing of those HCWs, nor can HCWs be well without the provision of such high-quality care. Some participants articulated that interrelationship, and the role QI may play in improvement. Others implied that they considered these were separate and isolated concepts.	‘*We need culture change that QI and wellbeing support for staff are essential to providing excellent patient care and improving staff wellbeing which includes positive flow on effects for recruitment and retention*’. p12 Doctor ‘*Lower level initiatives such as mentoring and social groups can help to cope with the stressors of work—but we need to address the base cause of poor wellbeing which is the overloaded healthcare system*’. p30 Doctor ‘*Wellbeing is often seen as a* ‘*dirty*’ *word or used in jest, and it is creating a culture that welcomes it as the bigger picture that is important—quality improvement projects, debriefing, safe staff spaces, staff safety—physically and psychologically*’ p13 Doctor ‘*Focus more on supplying required resources to provide quality care than trying to treat stress and burnout by Mindfulness!*’ p22 Doctor

### Mixed methods analysis: integration

Quantitative and qualitative results and analyses are generally congruent throughout the study, and imply that care quality and HCW wellbeing are limited by the current capacity and capability for QI and workplace wellbeing. Analyses were merged [18]. Integration strengthened the impetus for *why* change may be required: participants describe the distress of HCWs who work in milieux that is quantified as well below best-possible standards. This is associated with limitations to providing optimal care, to improving care, and to being well. *What* change may be required is clarified through integration: specific domains upon which improvement may be focussed are numerous, and provided throughout the survey instruments, for example, strengthening consumer input in QI. Overall, greater capacity and capability for QI and workplace wellbeing are needed. Integration also enables a theory to be generated for *how* capacity and capability for QI and workplace wellbeing may affect care quality and HCW wellbeing: because care quality depends upon HCW wellbeing, and HCW wellbeing depends upon providing high-quality care, the interdependence of HCW wellbeing and high quality care is highlighted. It follows that improvement in one may require improvement in the other, and a mechanism by which to improve each is by maximizing capacity and capability for *both* QI and workplace wellbeing.

## Discussion

### Statement of principal findings

This study was a mixed methods assessment determining the capacity and capability for QI and workplace wellbeing in NZ EDs. Quantitative analyses demonstrated considerable gaps between reported and best-possible capacity and capability for QI and workplace wellbeing, with the magnitude of results being approximately half of what may be considered ideal. Qualitative analysis identified three key themes: (i) HCW distress, (ii) HCW efforts to improve wellbeing, and (iii) interdependence of care quality and HCW wellbeing. Given the distress and wasted efforts of HCWs working in this suboptimal milieux, greater capacity and capability for QI and workplace wellbeing are required, which may improve the interdependent outcomes of care quality and HCW wellbeing.

### Strengths and limitations

Consistent with all single cross-sectional assessments, this study represents a point in time, and conclusions must be limited to associations. Because participants represent the majority of NZ Eds, the findings can reasonably be considered representative of EDs throughout NZ. However, generalizing findings outside that context requires caution.

Characteristics of participants represent strengths and limitations. Participants were anonymous, and most were members of the two largest ED workforce groups (doctors and nurses). The cohort was a relatively senior, experienced group, and self-identified as having expertise in quality and/or wellbeing. Their views may not represent the views of other NZ ED HCWs, the sample size is small, and nonrandom recruitment may be considered limitations. Additionally, some EDs were not represented by two participants as intended.

Rather than objective evidence, participant opinions were sought. The lack of a valid, reliable survey tool is an important limitation. Several survey instruments were used, each requiring modifications to ensure that questions were contextually appropriate. Modifications were led by a single, insider researcher, discussed with other research team members, and the survey was piloted by end-users. Results from the multiple instruments used were generally consistent.

The mixed-methods integration of quantitative and qualitative analyses strengthens the study by enabling a deeper understanding of the experience of NZ ED HCWs. Although assessing capacity and capability for both QI and workplace wellbeing together added complexity to the research, the approach is a strength because these can be considered interdependent.

### Interpretation within the context of the wider literature

Previous studies have assessed capacity and capability for either QI [11, 14] or HCW wellbeing [22]. To our knowledge, ours represents the first to solely assess capacity and capability for both QI and HCW wellbeing in the same mixed-methods study. Prior publications have described the use of QI to improve HCW wellbeing [8, 23] and the importance of considering HCW wellbeing in care quality [1] and QI [24]. To our knowledge, this is the first study in the NZ context to consider these associations.

Aside from the IR scale, there is scant reported use in the peer-reviewed literature of the study assessment tools, limiting comparison with other research. Original use of IR scale in the assessment of a single healthcare organization (*n* = 10 154 employees in 396 work settings) found an IR scale mean of 4.08 (SD 0.97) [14] and a mean positive IR climate score (4 or more) of 77.28% (SD 12.74). IR results from our study are considerably lower.

Previous assessments of QI capacity and capability in NZ healthcare concluded that QI in NZ healthcare is challenged by variable clinical staff engagement, inadequate resourcing of small internal QI teams, and the lack of focus on building capability [25]. Relating to wellbeing, a multilevel, whole-of-health system approach has recently been proposed, with practices planned nationally, coordinated regionally and delivered locally according to local needs [22].

There are calls for more quality fellowships to strengthen QI and its role in EDs [26]. ACEM have recently mandated QI training for EM trainees. Canadian ED HCWs with expertise in QI and workplace wellbeing recently made several recommendations, including that QI is best approached with a wellbeing lens [24].

Focussing at the ‘unit’-level to improve workplace wellbeing in healthcare organizations is exemplified at Stanford in the USA [23]. Unit-level wellbeing teams identify locally relevant problems, and work alongside locally led QI teams to improve using QI methods. Work is coordinated by an overall organizational wellbeing leader, and unit efforts are compared throughout the organization using maturity criteria.

East London NHS Foundation Trust in the UK reports use of the IHI Joy in Work Framework [27] to employ QI methods to improve staff and consumer wellbeing [28]. The Royal College of Psychiatrists Enjoying Work Collaborative reports several successes using a similar approach, including a reduction in HCW burnout [29]. Safer Care Victoria in Australia is using the IHI Framework [27] to partner with clinical teams, focus on the wellbeing of the workforce, build QI capacity and capability, and improve healthcare quality [30].

### Implications for policy, practice, and research

Policies and investments that facilitate structured and systematic growth of capacity and capability for QI and workplace wellbeing throughout the healthcare system are required. Supported at national and healthcare organization level, unit-level [23] led systematic improvements in areas identified as important to HCWs and consumers may effectively and efficiently improve HCW wellbeing and care quality. Locally relevant, valid, and reliable assessment tools are required for simpler more robust research.

## Conclusion

Considerable gaps were identified between reported and best-possible capacity and capability for QI and workplace wellbeing in NZ EDs. This is associated with HCW distress and wasted efforts. Policies that systematically increase capacity and capability for QI and workplace wellbeing may facilitate practice that can improve care quality and HCW wellbeing.

## Supplementary Material

mzaf073_Supplementary_Data

## Data Availability

Data are available from the lead author via email.
