# Supplementary material for: Quality improvement and workplace wellbeing capacity and capability in Aotearoa New Zealand emergency departments. A nationwide mixed methods survey
Source: Int J Qual Health Care. 2025 Aug 6;37(4):mzaf073. doi: 10.1093/intqhc/mzaf073 (PMC12500324; doi:10.1093/intqhc/mzaf073)
Supplement: mzaf073_Supplementary_Data [file mzaf073_supplementary_data.zip › 5.pdf]

Participant Info Consent

Thank you for participating in this survey.
This survey is to assess current capability for
quality improvement and
workplace wellbeing at your ED.
It is anonymous. It is possible that the lead investigator (Dr Mike Nicholls) may be able to identify you, but no one else will be able to.
We need two participants from each ED, one nursing and one medical, who are leaders of quality and workplace wellbeing at their ED.

This survey is part of a larger project aimed at improving the wellbeing of staff in NZEDs.
We hypothesise that:
by strengthening our ability to improve the quality of care we can provide, the wellbeing of staff in NZ EDs can be improved.

Below, please find attached the full participant information and consent form to download and read if you wish.

As a small token of thanks for your time and effort
there is up to 1Kg of coffee beans available to your department as koha...
at the end of the survey!
THANK YOU!

[Attachment: "Participant Information Sheet_Baseline Capability_QILS.docx"]

---

1) Would you like to proceed with the survey?
If you select "no", please "submit", then close the survey. Thank you

◯ Yes
◯ No

Demographic data

Some suggestions for the survey: Please use a computer (laptop or desktop), rather than a cellphone. The formatting on a cellphone may make it difficult to complete the survey We want 2 respondents (1 nurse, 1 doctor) from each ED, please. Leaders within the fields of workplace wellbeing and quality at their ED.
Please encourage an appropriate colleague from your ED to also participate You may not be sure of the answers to some of these questions That is ok, please give your best answer If you require, please consult with other colleagues in your ED who may have specialist knowledge in some areas The ED team is "inclusive of all people working in their respective roles within the ED environment" (ACEM quality standards definition)

This survey will take between 15-45 minutes You may save your responses and return to them later. You will be asked for your email address. There is no way for the researchers to find your email address. Please complete the survey by Friday 16th September Would you like to further contribute to the discussion and research on workplace wellbeing in NZ emergency departments? Following this survey, our next research step will be gathering more "rich" data from interviews of ED staff from 4 NZ EDs. If you may be available to participate in a 30-60 minute interview to further discuss these issues, we would be keen to hear from you. Please contact one of our researchers to discuss this.
Mike Nicholls. mnicholls@adhb.govt.nz Natalie Anderson na.anderson@auckland.ac.nz

---

2) Which ED do your answers apply to.

Please choose one only

- ◯ Ashburton Hospital
- ◯ Auckland City Hospital
- ◯ Bay of Islands Hospital
- ◯ Christchurch Hospital
- ◯ Dunedin Hospital
- ◯ Gisborne Hospital
- ◯ Grey Base Hospital
- ◯ Hawera Hospital
- ◯ Hawkes Bay Hospital
- ◯ Hutt Valley Hospital
- ◯ Kaitaia Hospital
- ◯ Lakes District Hospital
- ◯ Middlemore Hospital
- ◯ Nelson Hospital
- ◯ North Shore Hospital
- ◯ Oamaru Hospital
- ◯ Palmerston North Hospital
- ◯ Rotorua Hospital
- ◯ Southland Hospital
- ◯ Starship
- ◯ Taranaki Base Hospital
- ◯ Taumarunui
- ◯ Taupo Hospital
- ◯ Tauranga Hospital
- ◯ Thames Hospital
- ◯ Timaru Hospital
- ◯ Tokoroa Hospital
- ◯ Waikato Hospital
- ◯ Wairarapa Hospital
- ◯ Wairau Hospital
- ◯ Waitakere Hospital
- ◯ Wellington
- ◯ Whakatane Hospital
- ◯ Whanganui Hospital
- ◯ Whangarei Hospital
- ◯ Other...

---

3) What is your gender?

- ☐ male
- ☐ female
- ☐ another gender

4) Age group

◯ < 25
◯ 25-34
◯ 35-44
◯ 45-54
◯ 55-64
◯ >65
◯ Prefer not to answer

5) Ethnicity
Choose as many as appropriate

☐ NZ European
☐ Other European
☐ Māori
☐ Pacific peoples
☐ Asian
☐ Other
☐ Prefer not to answer

6) For Other

____

	< 1	1-5	6-10	11-15	16-20	>20
7) Years of emergency department work experience	◯	◯	◯	◯	◯	◯
8) Estimated number of improvement projects you have been involved with	◯	◯	◯	◯	◯	◯
9) Estimated number of improvement projects related to workplace wellbeing you have been involved with	◯	◯	◯	◯	◯	◯

10) What is your profession

◯ Nurse
◯ Doctor
◯ Other

11) Please outline any leadership roles you have held while working in EDs.

Baseline Assessment of New Zealand Emergency Department Capability for Quality Improvement and Workplace Wellbeing

The following questions ask about various aspects of Quality and Quality Improvement in your ED.

The questions are from

Institute for Healthcare Improvement Improvement Capability Self-Assessment Tool,

The Improvement Readiness Scale

ACEM Quality Standards and Implementation Toolkit

For each of the first 6 pages, please choose one answer per question.
The information in the tables provide some explanation about what each rating means.

Please choose which of these best describes the capability of the leadership of your department to:
set clear improvement goals, expectations, priorities, and accountability and to integrate and support the necessary improvement activities within the ED.

◯ Just Beginning ◯ Developing ◯ Making Progress ◯ Significant Impact ◯ Exemplary

Just Beginning
Developing
Making Progress
Significant Impact
Exemplary
There are no clear departmental level improvement goals, expectations, and priorities.
The department leadership has set clear improvement goals, expectations, and priorities through discussions with the ED team
ED leadership has prioritized some department level improvement goals to actively monitor and support.
Departmental leadership is actively engaged in monitoring and supporting most ED-level improvement goals.
Departmental leadership is actively engaged in monitoring and supporting all improvement goals.

◯ Just Beginning ◯ Developing ◯ Making Progress ◯ Significant Impact ◯ Exemplary

Just Beginning
Developing
Making Progress
Significant Impact
Exemplary
Improvement is seen as an individual or small group responsibility rather than requiring overall departmental leadership. Leadership for improvement is not coordinated across the department.
Local improvers are held accountable for achieving the established goals without the support required for them to bring about improvement. Department leadership does not fully facilitate improvement activities within the department.
Department leadership focuses on the system of care and supports some staff to facilitate coordination of improvement activities in the ED.
Departmental leadership focuses on the system of care and supports most staff in integrating and supporting improvement activities across the ED.
Department leadership focuses on the system of care and supports all staff in integrating and supporting improvement activities across the ED

◯ Just Beginning ◯ Developing ◯ Making Progress ◯ Significant Impact ◯ Exemplary

Just Beginning
Developing
Making Progress
Significant Impact
Exemplary
Very little, if any learning from improvement activities is shared across the department.
Some learning from improvement activities is shared across the department.
Department leadership has established a system for sharing the learning from some improvement activities across the ED.
Department leadership has established a system for sharing the learning from most improvement activities across the ED.

Department leadership has established a system for sharing the learning from all improvement activities across the ED.

If you wish to clarify any answers please do so.

Please choose which of these best describes your department to:
demonstrate measureable improvement across ED measures

◯ Just Beginning ◯ Developing ◯ Making Progress ◯ Significant Impact ◯ Exemplary

Just Beginning
Developing
Making Progress
Significant Impact
Exemplary
The ED can demonstrate measureable improvement, but this is not sustained over time and no sustained improvement can be demonstrated in any ED level measures.
Although some programs in the ED can demonstrate sustained and measureable improvement over time, very few if any of the ED-wide measures can demonstrate improvement over time.
The department has demonstrated sustained improvement over time for a few whole of ED measures.
The ED has demonstrated sustained improvement over time for most whole- of-ED measures.
The ED can demonstrate sustained improvement over time for all whole-of-ED measures.

Attached is a list of potential NZ ED measures. These were suggested as mandatory in a 2014 document.
You may wish to download this to clarify these.

[Attachment: "Summary of Mandatory ED Measures 2014 NZED.pdf"]

If you wish to clarify any answers please do so.

Please choose which of these best describes your department to provide sufficient resources to establish improvement teams within the department and to support their ongoing work and success.

◯ Just Beginning ◯ Developing ◯ Making Progress ◯ Significant Impact ◯ Exemplary

Just Beginning
Developing
Making Progress
Significant Impact
Exemplary
Resources are available within only a few programs or projects to support the work of improvement teams in these areas.
Resources are available within most programs or projects to provide adequate support to improvement activities focused in these areas.
Resources are available to support a coordinated approach to improvement across a number of programs or projects.
Resources are available to support improvement activities coordinated across most of the department
Resources are available to support and promote improvement activities coordinated across the whole department

◯ Just Beginning ◯ Developing ◯ Making Progress ◯ Significant Impact ◯ Exemplary

Just Beginning
Developing
Making Progress
Significant Impact
Exemplary
There is no department-wide coordination of resource allocation.
Some processes for allocating resources within the department have been established, but these are not coordinated across the department.
Some processes for allocating resources across the department are in place, but these are not fully coordinated across the department.
Some processes are in place to review and coordinate the allocation of resources for improvement across the department
Clear processes are in place to regularly review, prioritise, and coordinate the allocation of resources for improvement across the department

If you wish to clarify any answers please do so.

Please choose which of these best describes the capability of your department to organize its workforce to encourage and reward active participation in improvement work, clearly define and establish improvement leadership roles, and ensure that job descriptions include a component related to improvement work

◯ Just Beginning ◯ Developing ◯ Making Progress ◯ Significant Impact ◯ Exemplary

Just Beginning
Developing
Making Progress
Significant Impact
Exemplary
A few work groups (e.g. senior doctors, senior nurses) have identified a person who is responsible for improvement work.
Most work groups have identified improvement personnel, but they do not report directly to senior department leadership.
A plan for a clear chain of improvement accountability, responsibility, and leadership across the department has been developed.
All work groups have access to personnel who are responsible for improvement activities. The personnel have sufficient seniority to facilitate the changes required for improvement.
The department has established clearly defined improvement leadership roles.

◯ Just Beginning ◯ Developing ◯ Making Progress ◯ Significant Impact ◯ Exemplary

Just Beginning
Developing
Making Progress
Significant Impact
Exemplary

All staff see quality improvement as an integral part of their everyday work.

◯ Just Beginning ◯ Developing ◯ Making Progress ◯ Significant Impact ◯ Exemplary

Just Beginning
Developing
Making Progress
Significant Impact
Exemplary

The department encourages and rewards active participation in improvement work, and job descriptions include a component related to improvement work.

Please choose which of these best describes the capability of your department to establish, manage, and analyse data for improvement in a timely and routine manner to meet the objectives and expected results of the department's improvement plan

◯ Just Beginning ◯ Developing ◯ Making Progress ◯ Significant Impact ◯ Exemplary

Just Beginning
Developing
Making Progress
Significant Impact
Exemplary
The department uses data to measure performance, but only a few staff, groups or projects use data to support and inform improvement activities
The department uses data to measure performance and to support some improvement work
The department uses data to measure performance and to support most improvement projects
The department uses data to measure performance and to support almost all improvement projects
The department uses data to drive all improvement measures at the whole-of-ED level and in all improvement projects

◯ Just Beginning ◯ Developing ◯ Making Progress ◯ Significant Impact ◯ Exemplary

Just Beginning
Developing
Making Progress
Significant Impact
Exemplary
There is limited ability to communicate information across data management systems
The department is aware of a need to establish effective data management systems to communicate across key stakeholders and partners
The department has established/has capability with a number of data management systems to allow for some cross-system measures
The department has established/has capability with a number of data management systems which it uses routinely to share whole of ED performance information across key partners and stakeholders
Data management systems allow for highly effective communication within and across the department and with key stakeholders in a manner that informs the knowledge and actions required to meet the objectives of improvement teams.

If you wish to clarify any answers please do so.

Please choose which of these best describes the capability of your department to obtain and execute on the skills and competencies required to undertake improvement throughout the department

◯ Just Beginning ◯ Developing ◯ Making Progress ◯ Significant Impact ◯ Exemplary

Just Beginning
Developing
Making Progress
Significant Impact
Exemplary
Few if any quality improvement projects are under way that are guided by a department-wide improvement framework and model
A number of quality improvement projects are underway
A number of quality improvement projects have achieved measureable improvements
A number of quality improvement projects have achieved sustained improvement
The ED has embedded quality improvement in all areas of the department. Teams have achieved and sustained measureable improvements

◯ Just Beginning ◯ Developing ◯ Making Progress ◯ Significant Impact ◯ Exemplary

Just Beginning
Developing
Making Progress
Significant Impact
Exemplary
The hospital/department provides training in improvement methods to ED staff in a limited fashion
Multidisciplinary teams are formed and actively engaged

The department spreads learning from quality improvement projects systematically across the ED
The ED consistently shares and spreads improvements across the ED and with key stakeholders

If you wish to clarify any answers please do so.

Please rate your ED for the following standards.
0= ED does not fulfill the standard at all.
50 = ED partially fulfills this standard.
100 = ED fulfils this standard.
Please note, the ED team is inclusive of all people working in their respective roles within the ED environment.

The ED team utilises Māori health models (e.g. the Meihana Model) to inform clinical practice, to support ED staff to gain a broader understanding of Māori patients' presentations, and guide clinical assessment and treatment/intervention with Māori clients and whānau

ED does NOT fulfil this standard	ED PARTIALLY fulfills this standard	ED DOES fulfill this standard

(Place a mark on the scale above)

The composition of the ED team supports the necessary clinical and clinical support functions of the ED, including quality assurance, education provision, administration, research, maintenance, security, cleaning

ED does NOT fulfill this	ED partially fulfills this	ED does fulfill this

(Place a mark on the scale above)

The ED team engages with hospital administration and other hospital departments to enhance communication and accountability, and improve quality, safety, and efficiency of care

ED does NOT fulfill this	ED partially fulfills this	ED fulfills this

(Place a mark on the scale above)

The ED team has established actions to work collaboratively with hospital executive and inpatient units

ED dose not fulfill this	ED partially fulfills this	ED fulfills this

(Place a mark on the scale above)

The ED team has access to consumer representatives to engage in quality assurance and improvement activities

ED does not fulfill this	ED partically fulfills this	ED fulfills this

(Place a mark on the scale above)

The ED team demonstrates a capacity to learn from everyday successes as well as from adverse events

ED does not fulfill this	ED partially fulfills this	ED fulfills this

(Place a mark on the scale above)

If you wish to clarify any answers please do so.

Staff are empowered and supported to lead

ED does not fulfill this	ED partially fulfills this	ED fulfills this

(Place a mark on the scale above)

The ED team ensures each team member has the opportunity to debrief following a complex or stressful situation

ED does not fulfil this — ED partially fulfills this — ED fulfills this

(Place a mark on the scale above)

Debriefing processes address clinical and emotional issues

ED does not fulfill this — ED partially fulfills this — ED fulfills this

(Place a mark on the scale above)

The ED team ensures that the provision of inter-professional learning opportunities results in consistent work practices and expectations amongst the ED team

ED does not fulfill this — ED partially fulfills this — ED fulfills this

(Place a mark on the scale above)

The ED team has access to relevant non-technical skills training that enables the ED team to provide high quality care to patients including
communication skills, graded assertiveness, human factors, patient safety, continuous quality improvement, teamwork

ED does not fulfill this — ED partially fulfills this — ED fufills this

(Place a mark on the scale above)

If you wish to clarify any answers please do so.

The ED team has a process to systematically review and implement research and evidence-based findings relevant to the ED

ED does not fulfill this — ED partially fulfills this — ED fulfills this

(Place a mark on the scale above)

All members of the ED team are encouraged and empowered to participate in or contribute to quality improvement

ED does not fulfill this — ED partially fulfills this — ED does fulfill this

(Place a mark on the scale above)

Training and resources are provided to the ED team to conduct quality improvement

ED does not fulfill this — ED patially fulfills this — ED does fulfill this

(Place a mark on the scale above)

Patient involvement in quality improvement processes is encouraged and supported

ED does not fulfill this — ED partially fulfills this — ED does fulfill this

(Place a mark on the scale above)

If you wish to clarify any answers please do so.

Please tick which best describes your ED for the following attributes, beginning with the statement

"The improvement environment in this work setting...

	1. Disagree strongly	2.	3. Neutral	4.	5. Agree strongly
... utilises input/suggestions from the people who work here"	○	○	○	○	○
... integrates lessons learned from other work settings"	○	○	○	○	○
... effectively fixes defects to improve the quality of the work we do"	○	○	○	○	○
... allows us to gain important insights into what we do well"	○	○	○	○	○
... is protected by our local management"	○	○	○	○	○

If you wish to clarify any answers please do so.

How well integrated with the hospital Performance or Quality Improvement team is your ED team?

Not at all integrated	some integration	seamless, excellent integration

(Place a mark on the scale above)

What formal QI training have you had?

Please tick the number of courses for the following:

	0	1	2	3	>3
HQSC courses	○	○	○	○	○
IHI courses	○	○	○	○	○
university course	○	○	○	○	○
other courses	○	○	○	○	○

If you wish to clarify any answers please do so.

If there is anything related to QI capability not covered that you wish to comment upon, or anything else you wish to write, please do so.

Baseline Assessment of New Zealand Emergency Department Capability for Quality Improvement and Workplace Wellbeing

The rest of the survey relates to workplace wellbeing. The questions are from:
Wellbeing 2.0 table (Shanafelt) American Medical Association Joy in Medicine Health System Recognition Program ACEM Quality Standards and Implementation Toolkit
Please tick which best describes your ED for the following attributes related to workplace wellbeing.

Your ED may fall somewhere between one of these options. In that case choose 2 or 4.

Awareness state

◯ 1. Lack of awareness: inattention to the impact of ED staff distress
◯ 2.
◯ 3. Awareness: appreciation of the implications of ED staff distress and wellbeing
◯ 4.
◯ 5. Action: system interventions to prevent ED staff distress
◯ Not applicable/other

Professional culture

◯ 1. Culture of perfection
◯ 2.
◯ 3. Culture of wellness
◯ 4.
◯ 5. Culture of vulnerability and self-compassion
◯ Not applicable/other

Professional icon

◯ 1. ED staff with deity(god)-like qualities
◯ 2.
◯ 3. ED staff with hero-like qualities
◯ 4.
◯ 5. ED staff with human qualities
◯ Not applicable/other

Training mindset

◯ 1. "Rites of passage"
◯ 2.
◯ 3. Competency-based framework
◯ 4.
◯ 5. Organizational environment that attends to both developing competence and holistically caring for ED staff
◯ Not applicable/other

Please feel free to comment upon or clarify any answers

For the next 3 questions, there may be more than one answer.
Please tick as many that apply to your ED for the following attributes related to workplace wellbeing.

Organizational mindset - a focus on:

- ☐ institutional needs
- ☐ patient needs
- ☐ needs of people (patients, whānau and staff)
- ☐ Not applicable/other

Organizational mindset - towards workplace wellbeing:

- ☐ Individuals
- ☐ Teams
- ☐ Systems
- ☐ Not applicable/other

Organizational mindset - to solutions:

- ☐ Self-care
- ☐ Personal resilience
- ☐ Infrastructure and leadership to advance well-being
- ☐ Not applicable/other

If you wish to comment upon or clarify any answers please do so.

Please tick which one answer best describes your ED for the following attributes related to workplace wellbeing.

Your ED may fall somewhere between one of these options. In that case choose 2 or 4.

Organizational mindset

◯ 1. Blame individuals for distress (personal weakness)
◯ 2.
◯ 3. Appreciation that system factors cause distress, but promulgate personal solutions
◯ 4.
◯ 5. Address system issues through human factors engineering; evolve characteristics of organizational culture that contribute to distress
◯ Not applicable/other

ED staff - administrator relationship

◯ 1. Mutual neglect
◯ 2.
◯ 3. Adversarial: ED staff blame administrators for the problems; administrators blame staff
◯ 4.
◯ 5. ED staff-administrator partnership to create solutions
◯ Not applicable

ED staff mindset

◯ 1. No limits on work
◯ 2.
◯ 3. Balance personal and professional life
◯ 4.
◯ 5. Integration of personal and professional life including boundaries and healthy limits
◯ Not applicable

Administrator mindset

*organizations that view the issue as a zero-sum game problem have a mindset that the only way to relieve ED staff from excessive workload and administrative burden is to shift this work to others. This framework suggests that system approaches to improve well-being of some ED staff would invariably worsen the well-being of other members of the health care team.
** in a non-zero-sum game, all parties can gain.

◯ 1. Disregard of ED staff distress
◯ 2.
◯ 3. "Zero-sum game" problem*
◯ 4.
◯ 5. "Non-zero-sum game" problem**
◯ Not applicable

Funders (payers) and regulators mindset

◯ 1. No attention to impact that regulations and administrative decisions have on ED staff
◯ 2.
◯ 3. Awareness of impact that regulations and administrative decisions have on ED staff
◯ 4.
◯ 5. Regulations and administrative decisions influenced by needs of ED staff
◯ Not applicable

Orientation to colleagues

◯ 1. Isolation
◯ 2.
◯ 3. Connection
◯ 4.
◯ 5. Community (shared experience; care for each other, mutual support)
◯ Not applicable/other

Approach to individual distress

◯ 1. Neglect/ignore
◯ 2.
◯ 3. Treat
◯ 4.
◯ 5. Prevent distress and promote professional fulfillment
◯ Not applicable/other

Approach to technological contributions

◯ 1. Not applicable/lower relevance
◯ 2.
◯ 3. Teach ED staff tips and tricks to optimize their ability to use suboptimal IT
◯ 4. Demand better IT products from vendors
◯ 5. Collaborate with IT and regulatory bodies to limit suboptimal IT
◯ Not applicable

Scholarship and research focus

◯ 1. Rare descriptive studies of burnout/similar in some ED groups
◯ 2.
◯ 3. Limited testing of individual interventions to mitigate distress
◯ 4.
◯ 5.Rigorous testing of system-level interventions to mitigate distress and to promote staff wellbeing
◯ Not applicable

Resource allocation mindset regarding staff wellness

◯ 1. Ignorance of the issue
◯ 2. Staff well-being is a necessary cost
◯ 3. Return on investment
◯ 4. Value on investment
◯ 5. Staff well-being is a foundational value and core organizational strategy
◯ Not applicable/other

Please feel free to comment upon or clarify any answers

For the next 5 questions, there may be more than one answer.
Please tick as many boxes that apply to your ED.

Commitment

☐ formalised wellbeing group/committee
☐ position (< 0.5 FTE) devoted to wellbeing
☐ position (>=0.5 FTE) devoted to wellbeing
☐ formal strategic aim to improve wellbeing
☐ none of these

Assessment

☐ Burnout assessment within the last 3 years
☐ Burnout assessment every 12-24 months (at least two assessments)
☐ Burnout results shared with department leadership, and future targets established
☐ Estimate costs of burnout to department and shared with department leadership
☐ none of these

Leadership

☐ assessment of leadership skills for all leaders in past 24 months
☐ annual assessment of leaders
☐ implement leader development program
☐ tailor leadership development program based upon leadership assessments
☐ none of these

Teamwork

☐ measure teamwork within the last 3 years (with a validated tool such as Safety Attitudes Questionnaire)
☐ develop intervention based upon teamwork assessment
☐ none of these

Support

☐ peer support program to deal with adverse events
☐ peer support program, deal with broader issues beyond adverse events
☐ structured program to actively cultivate community at work
☐ none of these

Please feel free to comment upon or clarify any answers

Please rate your ED for the following standards.
0= ED does not fulfill the standard at all.
50 = ED partially fulfills this standard.
100 = ED fulfils this standard.
Please note, the ED team is inclusive of all people working in their respective roles within the ED environment.

The ED is designed to promote a positive environment for the ED team, patients, and whānau/carers, embracing cultural values of local communities.

ED does not fulfill this | ED partially fulfills this | ED does fulfill this

(Place a mark on the scale above)

The ED utilises good work design, where hazards and risks are removed or minimised and the wellbeing of workers is prioritised. Optimally, risks to workforce health and wellbeing are "designed out" of the work, where possible

ED does not fulfill this | ED partially fulfills this | ED does fulfill this

(Place a mark on the scale above)

Adequate facilities are provided for staff including change rooms, showers and toilets, secure storage, meal or break areas, office space, areas for group education, areas for breastfeeding, rest, reflection, prayer.

ED does not fulfill this | ED partially fulfills this | ED does fulfill this

(Place a mark on the scale above)

The ED team is involved in any investigation of increased turnover, including conducting exit interviews.

ED does not fulfill this | ED partially fulfills this | ED does fulfill this

(Place a mark on the scale above)

Turnover is minimised by system level interventions aimed at improving workforce experience

ED does not fulfill this | ED partially fulfills this | ED does fulfill this

(Place a mark on the scale above)

Please feel free to comment upon or clarify any answers

Occupational Health and Safety (OHS) representatives are supported and easy to contact.

ED does not fulfill this | ED partially fulfills this | ED does fulfill this

(Place a mark on the scale above)

Psychological safety is supported as well as physical safety.

ED does not fulfill this | ED partially fulfills this | ED does fulfill this

(Place a mark on the scale above)

The ED team advocates for the ED to be a safe and secure environment for team members.

ED does not fulfill this | ED partially fulfills this | ED does fulfill this

(Place a mark on the scale above)

The ED team ensures team members have access to evidence-based counselling, for work related stresses.

ED does not fulfill this | ED partially fulfills this | ED does fulfill this

(Place a mark on the scale above)

The ED team has systems in place where staff are regularly consulted in relation to how work is conducted, and departmental health and safety issues.

ED does not fulfill this | ED partially fulfills this | ED does fulfill this

(Place a mark on the scale above)

The ED has systems in place to identify, assess and control both physical and psychological risks of harm in the workplace.

ED does not fulfill this | ED partially fulfills this | ED does fulfill this

(Place a mark on the scale above)

There are processes in place for identifying, assessing and controlling physical, psychological and psychosocial hazards and risks e.g.
fatigue, high workloads, emotional demands, traumatic events, occupational violence, bullying and harassment, manual tasks.

ED does not fulfill this | ED partially fulfills this | ED does fulfill this

(Place a mark on the scale above)

The ED has a wellbeing policy which reflects support for the ED team as a priority

ED does not fulfill this | ED partially fulfills this | ED does fulfill this

(Place a mark on the scale above)

Please feel free to comment upon or clarify any answers

The ED team monitors absences to observe for signs of stress in the workforce.

ED does not fulfill this | ED partially fulfills this | ED does fulfill this

(Place a mark on the scale above)

Rosters comply with safe working hours recommendations.

ED does not fulfill this | ED partially fulfills this | ED does fulfill this

(Place a mark on the scale above)

Staff are able to access leave entitlements including
sick leave, parental leave, annual leave, professional development, carer's leave, leave for cultural obligations and celebrations.

ED does not fulfill this | ED partially fulfills this | ED does fulfill this

(Place a mark on the scale above)

The ED team has a healthy workplace plan which encourages the pursuit of health and wellbeing for team members. This includes providing education and training in achieving a healthy lifestyle and participating in preventative and health improvement initiatives

ED does not fulfill this | ED partially fulfills this | ED does fulfill this

(Place a mark on the scale above)

There are processes in place to monitor staff wellbeing, provide early intervention, and referral to appropriate support.

ED does not fulfill this | ED partially fulfills this | ED does fulfill this

(Place a mark on the scale above)

The ED team ensures that mentoring is available to all ED team members
separate from supervision or appraisal processes and is responsive to the needs of the mentee

ED does not fulfill this | ED partially fulfills this | ED does fulfill this

(Place a mark on the scale above)

ED leaders advocate for the psychological and physical safety and wellbeing of all team members, and work to address system factors that cause harm to the workforce.

ED does not fulfill this | ED partially fulfills this | ED does fulfill this

(Place a mark on the scale above)

Please list up to 3 things your team does related to workplace wellbeing that you consider noteworthy

Please list up to 3 things your team plans to do that may contribute to workplace wellbeing

The is the last question - except to get your coffee as Koha, next.

Is there anything related to Workplace Wellbeing capability not covered, or anything else you wish to write?

Would you like to further contribute to the discussion and research on workplace wellbeing in NZ emergency departments? Following this survey, our next research step will be gathering more "rich" data from interviews of ED staff from 4 NZ EDs. If you may be available to participate in a 30-60 minute interview to further discuss these issues, we would be keen to hear from you. Please contact one of our researchers to discuss this.
Mike Nicholls. mnicholls@adhb.govt.nz Natalie Anderson na.anderson@auckland.ac.nz

To find out information about the survey questions please click here.

Coffee as Koha

Thank you so much for your time, thought, and effort
Your responses are valuable.
We will continue to work hard to ensure that this research can contribute to further understanding, and improvement, of workplace wellbeing in EDs throughout Aotearoa.

As a gesture of thanks we would like to offer your department 1Kg of coffee beans from Allpress Coffee Allpress coffee.
We are keen to get 2 responses per department. One from the nursing team, one from the medical team.
Allpress will send
1Kg of coffee to each ED from which we receive 2 responses, or 250g to each ED from which we receive 1 response
So... on behalf of all coffee drinking members of your ED, please encourage your colleague to participate. Thank you.
As a default this will be addressed to the clinical director of the Emergency Deparment...please write if you'd prefer an alternative.
The mailing address of my department is:
