# Supplementary material for: Quality improvement and workplace wellbeing capacity and capability in Aotearoa New Zealand emergency departments. A nationwide mixed methods survey
Source: Int J Qual Health Care. 2025 Aug 6;37(4):mzaf073. doi: 10.1093/intqhc/mzaf073 (PMC12500324; doi:10.1093/intqhc/mzaf073)
Supplement: mzaf073_Supplementary_Data [file mzaf073_supplementary_data.zip › 6.docx]

Table 1. Workplace wellbeing measured by Joy in Medicine Health System Recognition Program^1^

| Commitment |  | n(%) |
| --- | --- | --- |
| B | formalised wellbeing group/committee | 19(32) |
| S | position (< 0.5 FTE) devoted to wellbeing | 7(12) |
| S | position (>=0.5 FTE) devoted to wellbeing | 3(5) |
| G | formal strategic aim to improve wellbeing | 7(12) |
| **N=59** | none of these | 31(53) |
| Assessment |  |  |
| B | Burnout assessment within the last 3 years | 7(12) |
| S | Burnout assessment every 12-24 months (at least two assessments) | 2(3) |
| S | Burnout results shared with department leadership, and future targets established | 3(5) |
| G | Estimate costs of burnout to department and shared with department leadership | 2(3) |
| **N=59** | none of these | 48(81) |
| Leadership |  |  |
| B | assessment of leadership skills for all leaders in past 24 months | 3(5) |
| S | annual assessment of leaders | 12(20) |
| S | implement leader development program | 8(14) |
| G | tailor leadership development program based upon leadership assessments | 0(0) |
| **N=59** | none of these | 39(66) |
| Teamwork |  |  |
| B | measure teamwork within the last 3 years (with a validated tool such as Safety Attitudes Questionnaire) | 3(5) |
| G | develop intervention based upon teamwork assessment | 2(3) |
| **N=59** | none of these | 54(92) |
| Support |  |  |
| B | peer support program to deal with adverse events | 18(31) |
| S | peer support program, deal with broader issues beyond adverse events | 14(24) |
| G | structured program to actively cultivate community at work | 7(12) |
| **N=59** | none of these | 29(49) |

Note, participants could check as many options as they felt appropriate. n=Number of participant responses; FTE= full time equivalent. B=Bronze, S= Silver, G= Gold criteria.

1. American_Medical_Association. Joy in Medicine Health System Recognition Program. 2022. ama-assn.org/amaone/practice-transformation.
