# Supplementary material for: Quality improvement and workplace wellbeing capacity and capability in Aotearoa New Zealand emergency departments. A nationwide mixed methods survey
Source: Int J Qual Health Care. 2025 Aug 6;37(4):mzaf073. doi: 10.1093/intqhc/mzaf073 (PMC12500324; doi:10.1093/intqhc/mzaf073)
Supplement: mzaf073_Supplementary_Data [file mzaf073_supplementary_data.zip › 4.docx]

Quality Improvement and Workplace Wellbeing Capacity and Capability in Aotearoa New Zealand Emergency Departments. A Nationwide Mixed Methods Survey

# Supplementary File 1. Development of Survey Questions

Quality Improvement in healthcare is challenging, and results of QI are often mixed.^1,2^ In addition to the content of any improvement initiative, how and within what context QI is implemented is important. An understanding of context, defined as "all factors that are not part of a QI intervention itself"^3^ when planning, implementing, evaluating and researching QI initiatives is widely acknowledged as vital to success in QI.^1,4-11^ An ideal tool to assess our ED setting would have been previously assessed for validity and reliability in NZ ED settings. To our knowledge no such tool exists.

Tools to assess capability to undertake QI exist.^11^ Some tools assess such capability at an organisational (hospital or healthcare organisation) level. Examples include, the Organisational Strategy for Improvement Matrix (OSIM) from Safer Care Victoria, Australia,^12^ Building the Foundations for Improvement checklist^13^ from UK, and the Institute for Healthcare Improvement (IHI) Improvement Capability Self-Assessment Tool^14^ from the US. The IHI has good face validity the IHI tools are ubiquitous throughout the NZ health care system, including the HQSC. The Improvement Readiness (IR) Scale^15^ is composed of only 5 statements to assesses organisational capability for QI at the departmental level by all staff at that workplace and is a promising tool for future use. Other available tools assess capability to implement a specific, individual project. Examples are the original or modifications of The Model for Understanding Success in Quality (MUSIQ),^6^ and Fitness for Improvement Tool (FFIT).^16^ Additional assessments may be based upon a particular improvement methodology, e.g. Lean,^7^ or assess the capability of individuals, e.g., Quality Improvement Knowledge Assessment Tool (QIKAT).^17^ Specific to NZ EDs, there is a checklist of quality indicators for NZ EDs,^18^as well as the ACEM Quality Standards Implementation Toolkit.^19^

In order to assess NZ ED QI capability we will use modified questions and statements from the IHI self-assessment tool, the IR scale, and the ACEM Quality Standards Implementation Toolkit.^14,15,19^

No robust tool exits to assess ED-level workplace wellbeing capability. We have based survey questions and statements upon the "era" of workplace wellbeing described by Shanafelt,^20^ the Joy in Medicine Health System Recognition Program ^21^ and the ACEM Quality Standards Implementation Toolkit.^19^

# Survey Questions

As noted, no ideal instrument existed that suited our objectives. Participants were asked about demographics, years of ED experience, and number of previous QI courses and workplace wellbeing and QI projects. Other survey questions were sourced from:

- the IHI self-assessment tool^14^
- the IR scale^15^
- the ACEM Quality Standards Implementation Toolkit^19^
- the "era" of workplace wellbeing described by Shanafelt,^20^
- the Joy in Medicine Health System Recognition Program ^21^

These were validated in several steps. To suit the purposes of the study the Principal Investigator (PI) modified the five documents from which the original questions were sourced. For example "*Māori*" replaced "First Nations People", "Emergency Department" replaced "Organisation", and "ED staff" replaced "Physicians". Some statements were excluded from a tool if they were not applicable to the project. For example statements related to the Electronic Health Record (EHR) in the Joy in Medicine Health System Recognition Program were excluded because they were deemed not relevant to NZ ED setting in which the EHR, while associated with frustrations, seems to be considerably different in NZ compared to US. Other authors were given an opportunity to alter, remove or add some questions or statements. The survey questions were then piloted by members of the executive team to check usability, relevance and understanding. Small alterations were made as a result, prior to being distributed to participants. The PI had the final decision about the structure and content of the survey.

Similarly, viewing organisational capacity and capability for workplace wellbeing with a 'maturity' lensis exemplified by Shanafelt who describes three '*eras*' of wellbeing, the Era of Distress, Well-being 1.0, and Well-being 2.0.^20^ The Joy in Medicine Health System Recognition Program^21^ also uses a maturity approach for assessment of HCW wellbeing at the organisation level.

## Quality Improvement Capability

### Institute for Healthcare Improvement Capability Self-Assessment tool.

The IHI tool has six "key areas" for assessment: leadership for improvement; results; resources; workforce and human resources; data infrastructure and management; and improvement knowledge and competence. Most areas translated easily from "organisation" to "department" level.

### Improvement Readiness Scale

A promising instrument for QI capability assessment at the department level is the Improvement Readiness (IR) Scale.^15^ First described in 2018 in a study of 10,627 multidisciplinary healthcare participants from 440 workplace settings in a single large US academic healthcare system, it is brief and simple. Composed of only 5 statements, each begin with "The learning culture in this work setting ..." followed by a statement related to improvement readiness, for example, "...utilises suggestions/ideas from the people that work here". Respondents use a 5-point Likert scale to rate their agreement with the statement. In the original study there was good internal reliability (Cronbach's alpha 0.93 for the overall sample), and the scale correlated well with measures of safety and burnout. Given the importance of minimising respondent burden the brevity of the scale is an attractive feature. This was considered a useful measure with which to survey all ED staff to assess organisational capability for QI in the future, particularly with further assessments of validity and reliability. This scale was used unmodified from the original.

### ACEM Quality Standards Implementation toolkit.

The ACEM toolkit is an 802 page online document that comprises five Domains and an additional overarching Cultural Safety section. The five domains are: clinical care patient pathway; administration; professionalism; education and training; and research. Three statements relate directly to QI in the Toolkit. These were from Standard 5.4 Quality Improvement in the Research Domain. Other statements were chosen from throughout the document. There were no modifications of these statements.

## Workplace Wellbeing Capability

### Eras of Wellbeing

Shanafelt describes 3 eras of wellbeing, with 14 "characteristics" of organisations. These characteristics include organisational mindset, approach to individual distress, and approach to technological contributions. Although originally aimed at the organisational level, physicians, and HCOs in USA, these were largely adopted with minimal changes. Most notable changes include "Physician" for "ED Staff" and "ED" or "department" for "organisation".

### Joy in Medicine Health System Recognition Program.

From American Medical Association (AMA), this has recently been developed to recognise organisations that are working towards system solutions to workplace wellbeing problems. Organisations are ranked as bronze, silver or gold organisations depending upon the maturity of their system responses to burnout. From the USA, this is aimed at organisations with more than 100 physicians or advanced practitioners. Although inclusion of all staff groups is required for more advanced ranking, physicians are the main focus. Modifications for the study to suit the NZ ED setting were made, for example, excluding reference to the "Electronic Health Record" which is a major source of frustration in US healthcare, but is better referred to in our study from other sources, e.g., ACEM Quality Standards.

### ACEM Quality Standards Implementation toolkit.

There were numerous statements that were relevant to the assessment of WoWe CC throughout this large document. Several statements have been included emphasis recent ED-specific developments. For example, mentoring has been targeted by ACEM over the last decade and has been considered particularly important for EM trainees. Given the centrality of the team in ED, mentoring applies to all members of the ED team, for example nurses and other non-clinical roles, e.g. clerical staff. Many statements excluded from the final survey could have been included. For example, Standard 2.1 / Built environment, Objective B / ED layout (*The ED is designed to ensure both patients and ED team members are safe and secure within the ED*) could have been included. However, a balance was struck between our aspirations for thoroughness and pragmatism to reduce respondent burden.

1. Davidoff F, Dixon-Woods M, Leviton L, Michie S. Demystifying theory and its use in improvement. BMJ Quality & Safety 2015;24(3):228-238. DOI: 10.1136/bmjqs-2014-003627.

2. Akmal A, Podgorodnichenko N, Foote J, Greatbanks R, Stokes T, Gauld R. Why is Quality Improvement so Challenging? Health Policy 2021;125(5). DOI: 10.1016/j.healthpol.2021.03.015.

3. Øvretveit J. Understanding the conditions for improvement: research to discover which context influences affect improvement success. BMJ Quality & Safety 2011;20(Suppl 1):i18-i23. DOI: 10.1136/bmjqs.2010.045955.

4. Reed JE, Kaplan HC, Ismail SA. A new typology for understanding context: qualitative exploration of the model for understanding success in quality (MUSIQ). BMC Health Services Research 2018;18 (Report) (In English) (<https://link.gale.com/apps/doc/A547773169/AONE?u=learn&sid=bookmark-AONE&xid=188656ec>).

5. Kaplan HC, Walsh KE. Context in Implementation Science. Pediatrics 2022;149(Suppl 3). DOI: 10.1542/peds.2020-045948c.

6. Kaplan HC, Provost LP, Froehle CM, Margolis PA. The Model for Understanding Success in Quality (MUSIQ): building a theory of context in healthcare quality improvement. BMJ Quality & Safety 2012;21(1):13-20. DOI: 10.1136/bmjqs-2011-000010.

7. Wilson WJ, Jayamaha N, Frater G. The effect of contextual factors on quality improvement success in a lean-driven New Zealand healthcare environment. International Journal of Lean Six Sigma 2018;9(2):199-220. (In English). DOI: <https://doi.org/10.1108/IJLSS-03-2017-0022>.

8. Rees GH. Organisational readiness and Lean Thinking implementation: Findings from three emergency department case studies in New Zealand. Health services management research 2014;27(1-2):1-9. DOI: 10.1177/0951484814532624.

9. Coles E, Anderson J, Maxwell M, et al. The influence of contextual factors on healthcare quality improvement initiatives: a realist review. Systematic reviews 2020;9(1):94-94. (In eng). DOI: 10.1186/s13643-020-01344-3.

10. Nielsen K, Miraglia M. What works for whom in which circumstances? On the need to move beyond the ‘what works?’ question in organizational intervention research. Human Relations 2017;70(1):40-62. DOI: 10.1177/0018726716670226.

11. Furnival J, Boaden R, Walshe K. Conceptualizing and assessing improvement capability: a review. International Journal for Quality in Health Care 2017;29(5):604-611. DOI: 10.1093/intqhc/mzx088.

12. Murray C. Organisational Strategy for Improvement Matrix (OSIM). Safer Care Victoria, , Melbourne., 2018. (<https://www.safercare.vic.gov.au/publications/organisational-strategy-for-improvement-matrix-osim>).

13. Jones B, Woodhead T. Building the foundations for improvement. How five UK trusts built quality improvement capability at scale within their organisations. The Health Foundation, 2015. (<http://www.health.org.uk/publications/building-the-foundations-for-improvement>).

14. IHI. IHI Improvement Capability Self‐Assessment Tool. Institute for Healthcare Improvement; 2014.

15. Adair KC, Quow K, Frankel A, et al. The Improvement Readiness scale of the SCORE survey: a metric to assess capacity for quality improvement in healthcare. BMC Health Services Research 2018;18(1):975. DOI: 10.1186/s12913-018-3743-0.

16. Wright D, Gabbay J, Le May A. Determining the skills needed by frontline NHS staff to deliver quality improvement: findings from six case studies. BMJ Quality & Safety 2021:bmjqs-2021-013065. DOI: 10.1136/bmjqs-2021-013065.

17. Singh MK, Ogrinc G, Cox KR, et al. The Quality Improvement Knowledge Application Tool Revised (QIKAT-R). Academic Medicine 2014;89(10):1386-1391. DOI: 10.1097/acm.0000000000000456.

18. National_Emergency_Departments_Advisory_Group. A Quality Framework and Suite of Quality Measures for the Emergency Department Phase of Acute Patient Care in New Zealand. In: Health. Mo, ed. Wellington2014.

19. ACEM. Quality Standards for Emergency Departments and Hospital-Based Emergency Care Services Toolkit. <https://acem.org.au/Content-Sources/Advancing-Emergency-Medicine/Better-Outcomes-for-Patients/Quality-Standards2022>.

20. Shanafelt TD. Physician Well-being 2.0: Where Are We and Where Are We Going? Mayo Clin Proc 2021;96(10) (<https://doi.org/10.1016/j.mayocp.2021.06.005>).

21. American_Medical_Association. Joy in Medicine Health System Recognition Program. (ama-assn.org/amaone/practice-transformation).
